# The effectiveness of job rotation to prevent work-related musculoskeletal disorders: protocol of a cluster randomized clinical trial

**DOI:** 10.1186/1471-2474-15-170

**Published:** 2014-05-22

**Authors:** Maria Luiza Caires Comper, Rosimeire Simprini Padula

**Affiliations:** 1 Masters and Doctoral Programs in Physical Therapy, Universidade Cidade de São Paulo, Rua Cesário Galeno 475, 03071-000 São Paulo, Brazil; 2 Discipline of Physical Therapy, União Metropolitana de Ensino e Cultura, Itabuna, Brazil

**Keywords:** Job design, Job rotation, Physical workload, Work related musculoskeletal disorders

## Abstract

**Background:**

Job rotation has often been used in situations where the level of exposure cannot be reduced due to the characteristics of the job or through physical measures. However, the effectiveness of the job rotation strategy at preventing musculoskeletal complaints lacks adequate scientific data.

**Methods/Design:**

A cluster randomized controlled trial will be used to investigate the effectiveness of job rotation to prevent musculoskeletal disorders in industrial workers. The randomized cluster was based in characteristics of production sectors. A total cluster will be 4 sectors, and 957 workers will be recruited from a textile industry and randomly allocated into intervention or control groups. Both groups will receive training on ergonomics guidelines. In addition, the intervention group will perform job rotation, switching between tasks with low, moderate, and high risk for musculoskeletal complaints. The primary outcome will be the number of working hours lost due to sick leave by musculoskeletal injuries recorded in employee administrative data bases. Secondary outcomes measured via survey include: body parts with musculoskeletal pain, the intensity of this pain, physical workload, fatigue, general health status, physical activity level, and work productivity. Secondary outcome measures will be assessed at baseline and after 3, 6, 9, and 12 months. The cost-effectiveness analysis will be performed from the societal and company perspective.

**Discussion:**

Prevention of work-related musculoskeletal disorders is beneficial for workers, employers, and society. The results of this study will provide new information about the effectiveness of job rotation as a strategy to reduce work-related musculoskeletal disorders.

**Trial registration:**

NCT01979731, November 3, 2013

## Background

Ergonomic interventions to improve working conditions and to prevent the occurrence of occupational diseases have been widely used in industrial production lines [1]. In case of productive cells characterized by tasks that require some type of physical burden and/or the adoption of cognitive interventions to reduce the level or duration of exposure of workers, such as: redesign of jobs, suitable furniture and tools, ergonomic guidelines, rest breaks and rotation function [2]. The latter, considered organizational measures are generally adopted in tasks whose exposure level cannot be lowered due to the characteristics of the job or through physical measures [3].

Job rotation is one of the most practiced organizational interventions, such as cost reduction and/or prevention and health promotion for workers. Cost reduction is achieved by training workers to perform a greater number of functions for flexible allocation of worker activity. The prevention and health promotion for workers occurs through switching between different tasks with different levels of exposure and biomechanical applications, which in theory reduce the cumulative and or average exposure that should in turn promote the reduction of musculoskeletal and cognitive overloads [2,4].Thus, the job rotation has been adopted in repetitive, static, or monotonous activities, aiming to relieve the effects of muscle and cognitive overload, monotony, absenteeism, and stress [5,6].

Regarding the assessment of the effectiveness of the job rotation and the prevention of musculoskeletal complaints strategy, studies show conflicting results. Hinnen et al. evaluated supermarket workers by a cross-sectional study and found a 40% reduction in complaints of neck pain and a 20% reduction in complaints of pain in the shoulder for those who carried out labor [7]. Another study compared groups who performed or did not perform job rotation, and also found significant reductions in physical burden on workers of a garbage collection department who underwent rotation between the driving task and collecting trash [8]. However, while performing in a longitudinal study, it was observed that in the long run, the job rotation increased overload on other body regions, and workers began to report more back pain, especially in the group who had just completed the driving task [9]. Probably, it is because the groups continued exposure to the same risk factors, even when switching the task. Similar results were obtained by Frazer et al. [10], who evaluated two tasks of material handling, with low and high overhead level. The risk of lower back pain increased as a greater amount of time was used to perform the task with higher overhead, due to the cumulative and peak force [10].

The different study methods and criteria used for deployment of job rotation may partly explain the heterogeneity of results. This, plus the absence of clinical, controlled, randomized studies evaluating the effectiveness of the job rotation and the prevention of musculoskeletal complaints strategy, complicates the clinical decision making of professionals in the health and safety of the worker.

Importantly, positive results for the worker's health will only be achieved if the planning of the job rotation meets some important criteria such as number of workers and tasks involved, exposure level, requested body region, frequency of movements, duration of exposure, and duration of rest break, among others [11]. To assist in this planning, some methods and algorithms have been developed using the variables mentioned before [11-14]; however, the proposed rotation as reported in the literature, has not been evaluated in controlled clinical studies or randomized, and the effect of reducing absenteeism caused by musculoskeletal disorders in the workplace is not yet proven.

All these limitations point to the need for implementing a well design job rotation program along with a well-designed intervention study in order to fully and robustly evaluate the theory of job rotation and its application. Hence, we aimed to develop such a well-designed job rotation program that has specific criteria to reduce the cumulative and average biomechanical exposure and then to evaluate the effect of this newly developed job rotation program in the prevention of musculoskeletal disorders in industrial textile workers.

## Methods

### Study design, approval and registration

This is a randomized cluster controlled trial, prospectively registered, and with blinded assessment will be used to investigate the effectiveness of job rotation to prevent musculoskeletal disorders in industrial workers. The procedures and consent form were approved by the Research Ethics Committee of Cidade de São Paulo University (protocol no. 18170313.5.0000.0064) and were prospectively registered at ClinicalTrials.gov – NCT01979731. The study is being funded by the National Counsel of Technological and Scientific Development (CNPq), Brazil (473651/2013-0).

### Study population and setting

Study participants will be production line workers, recruited from the textile industry of a large company in Bahia, Brazil. The productive sectors of this industry will be classified according to the level of exposure to risk factors for musculoskeletal pain and disorders. The sectors to be included should have production lines that allow switching between tasks with different biomechanical demands and levels of risk for musculoskeletal pain and disorders, and further, that the work is carried out in cell production. Sectors whose production lines are automated or semi-automated, possessing the pace of work determined by machinery where work stoppage is not possible will be excluded. All workers in selected sectors will be invited to participate in the study.

### Sample size calculation

**Figure 1:**
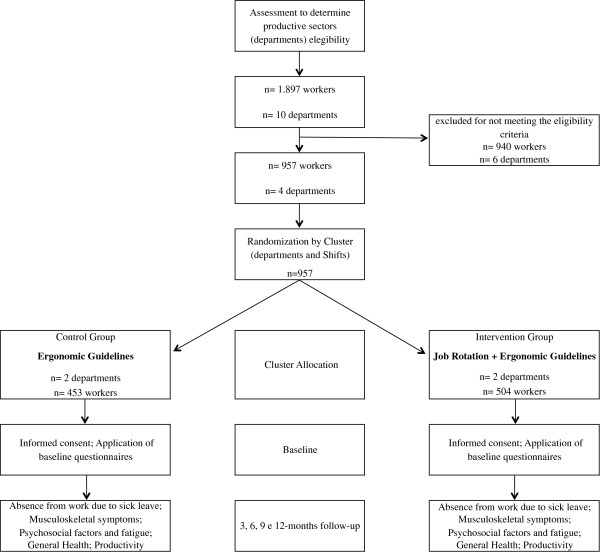
Study flow diagram.

The number of working hours lost due to sick leave by musculoskeletal injuries (M Group International Classification of Diseases, ICD-10) was used to estimate the sample size. The average time lost during the last three months in companies that the study observed was approximately 1,100 hours. The authors assume that interventions will enable a reduction of this number by 10%, that is, the groups will have a difference of 100 lost working hours, with a standard deviation of 250 hours. A statistical power of 80%, an alpha of 5%, and a possible sample loss of up to 15% is considered. Therefore, 116 participants are needed per group, or 232 in total. But, in this study the calculation of sample size was made by clusters and considered the number of production sectors included. The clusters number was four sectors (n = 957). The intervention group has 504 workers selected from Finishing Socks and Finishing Underwear departments, and the control group is 453selected from Sewing Socks and Sewing Underwear departments (Figure 1).

### Randomization

Randomization was performed at the group level (departments/productive sectors), in order to avoid contamination from workers allocated in the intervention to those in the control group. The productive sectors included in the study were pre-stratified by the level of exposure to risk factors for musculoskeletal pain and disorders. Sectors with similar demands were grouped and randomly divided between intervention and control groups. Using a computer-generated randomization (http://www.randomizer.org) with random numbers to define groups, the randomization was performed by an independent researcher who was not involved in recruitment and assessment. Sealed envelopes will be used. The randomization was performed before baseline measurements.

### Blinding

Obviously, the nature of the intervention used in this study made it impossible to blind workers and ergonomists. However, the workers’ interview researcher will be blind to the study design and the group assignment. To test blinding, after assessment of outcomes, the worker’s assessment researcher will note his opinion as to the type of intervention received by workers. Moreover, all workers in both groups will receive ergonomic guidelines, used as a placebo intervention.

### Study groups

#### ***Control group***

Workers allocated in the control group will be invited to attend training with ergonomic guidelines, taught by a physiotherapist/ergonomist. The training will be held on a single day over 4 hours, with lectures on: ergonomic risk factors and their influence on the development of musculoskeletal symptoms, improvements and adaptations for workstations, work postures, and preventive exercise [15]. Participants in the intervention group will receive the same information.

#### ***Intervention group***

Workers allocated in intervention sectors will receive the same ergonomic guidelines as the control group. In addition, they will realize job rotation, as described below.

### Intervention

#### ***Job rotation***

The job rotation is proposed in order to modify exposure, so that workers will perform tasks alternating between low, moderate, and high risk for musculoskeletal pain and disorders and exposure to risk for injury to different body regions. Therefore, an ergonomic analysis will indicate, based on the main posture adopted to carry out the task, the body regions of higher overload level and intensity of exposure. The autonomy of the work (techniques and work rate) and the use of machines and tools will also be evaluated. The level of exposure to risk of musculoskeletal pain and disorders will be assessed by Quick Exposure Check (QEC) [16,17] and Rapid Entire Body Assessment (REBA) [18]. These methods enable the characterization of worker exposure to the task because they assess the main risk factors for musculoskeletal disorders (frequency of movements and postures performed by spine and upper limbs, amount of weight handled, time to perform the task, manual force, visual demands of the activity, presence of vibration, work pace, and stress) [16,17].

**Figure 2:**
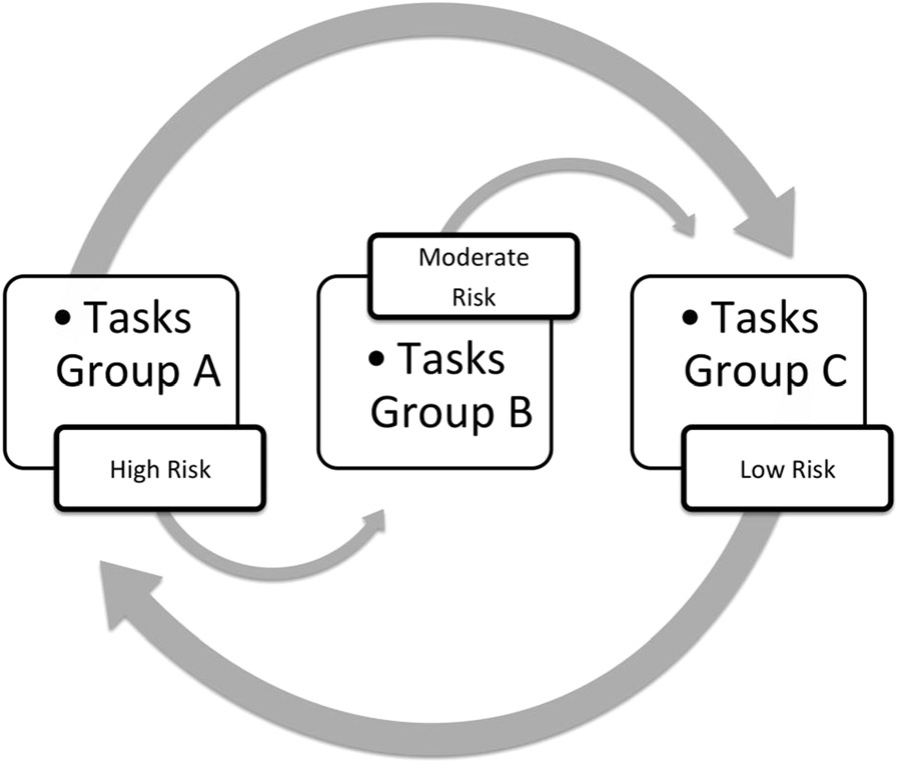
**Job rotation design.** Group A (operate Alome machine; Firsan- shaping socks; separation of product’s color and size; stamping; Tecma machine packaging; silicon preparing); Group B (Alome - choice of pair of socks; operate Satellite machine; operate Sealing machine; fix fast pin; automatic packaging); Group C (Firsan- choice of pair of socks; Satellite packing socks; Alome – socks review; manual packaging; operate Autotex machine; application of silicon; seamless products review).

The structure of the job rotation will be proposed from the results obtained by ergonomic assessment, according to the following priority criteria: (1) level of exposure intensity (low, moderate, high, or very high), (2) posture predominantly adopted for conducting task (sitting, kneeling, standing, walking), (3) the main physical demand (material handling, repetition of movements, static posture), and (4) body regions of higher overhead (shoulders, elbows, wrists, hands, spine) (Table 1). Tasks that have similarity of these criteria will be grouped to then be alternated, so that: (1) tasks with exposure level will be low or moderate risk tasks alternated with high and very high, (2) tasks that require a predominantly standing posture will alternate with tasks that require a sitting posture, (3) handling tasks will be alternated with tasks requiring repetition of movements, and (4) the tasks alternate body regions of higher overhead (Figure 2). The completion of the rotation will take place at intervals of 2 hours. It is estimated that this time is compatible with a lower lactic acid [19].

**Table 1:** Summarized description of the job rotation definition

**Step**	**Definition**	**Description**
1	**Ergonomic work analysis**	Evaluate the main demand of work posture adopted in the task, movements per body region (amplitude and frequency), autonomy, work rate, percentage of occupation in work cycles
2	**Ergonomic exposure risk level assessment**	Classify the activity exposure level ergonomic (low, moderate, high and very high) by two observational protocols: Quick Exposure Check (QEC) and Rapid Entire Body Assessment (REBA)
3	**Job rotation definition**	Propose the structure of job rotation in accordance with the following priority criteria: (1) the level of exposure intensity (low, moderate, high or very high), (2) predominantly stance taken by the completion task (sitting, kneeling, standing, walking), (3) the main physical demand (material handling, repetition of movements, static posture), (4) body regions of higher overhead (shoulders, elbows, wrists, hands, spine), (5) production’s specificities

### Assessment instruments and outcomes

Although the intervention is applied to the production sectors, the outcomes are on an individual level, since they reflect aspects of the health of workers. Data regarding outcome measures will be assessed at baseline and after 3, 6, and 12 months, except for information relating to removal for sick leave, which will be recorded daily. After a period of 12 months, the sector that had received only ergonomic guidelines will receive job rotation considering the hypothesis of this study in relation to its effectiveness. Both groups of workers will be monitored in a longitudinal study in subsequent months and compared to other workers identified within the criteria for risk analysis for 24 months.

### Socio-demographic

At baseline, socio-demographics (age, sex, level of education, length of service in the company and the current role, working days per week, and hours of work per week, among others) will be assessed using a questionnaire developed by the researchers of this study.

### Primary outcome measure

#### ***Absence from work due to sick leave***

In this study, we will use the sick leave for sick leave due to illness of the musculoskeletal system and connective tissue as a primary outcome measure. This outcome will be measured by the number of working hours lost due to the removal of a symptom or disease of the musculoskeletal system and connective tissue. These data will be obtained from records of medical certificates validated by the Human Resources department (HR) of each industry. Information regarding the clinical diagnosis and body region will also be collected using the specific codes of the International Classification of Diseases, 10th revision (ICD-10).

### Secondary outcome measure

#### ***Musculoskeletal symptoms***

The occurrence of musculoskeletal symptoms (pain, tingling, or numbness) will be assessed using the Nordic Musculoskeletal Questionnaire (QNSO) [20]. The respondents will answer simple questions (yes or no) in relation to musculoskeletal symptoms they have experienced in the past 12 months and/or in the past seven days, as well as regarding the occurrence of disability and demand for health aid professionals in the last 12 months due to these symptoms. The intensity of the pain will be assessed by the Verbal Numerical Pain Scale [21]. It is an 11-point scale, where 0 means "no pain" and 10 means "worst possible pain."

### Risk factors for musculoskeletal pain and disorders

Some studies show that ergonomic interventions reduce the occurrence of removal for medical leave due to lower levels of exposure to risk factors [22,23]. This study addresses the perception of workers against risk factors that may contribute to the development of musculoskeletal complaints through the Job Factors Questionnaire. This instrument presents a descriptive list of 15 risk factors that should be classified on a scale of zero to ten, indicating how much each factor contributed to the emergence of work-related musculoskeletal symptoms, with zero meaning "no problem" and ten indicating the "largest possible problem" [24].

### Psychosocial factors and fatigue

Data on the perception of workers against psychosocial factors and stress, resulting in fatigue induced by work, will be obtained through the Scale of Need for Recovery [25]. This Likert-type scale has 11 questions and possible answers of numbers up to 4 (0 = never, 1 = sometimes, 2 = often, and 3 = always). The answer “always” indicates an unfavorable situation and receives 3 scores, with the exception of item 4, which features reverse scoring. The total score is obtained by summing the final, transformed by rule of three direct, on a scale of 0 (minimum) to 100 (maximum). In this case, the higher the score, the greater the amount of higher symptoms and need of recovery.

### General health

The WHOQOL-BREF will be used to assess the overall health status and quality of life of workers. This instrument contains 26 questions, divided into four areas: social, psychological, physical, and environment. Each domain consists of questions whose answer scores range between 1 and 5 [26].

### Productivity

Besides this information, other single issues related to productivity at work will be answered by the workers during the follow-ups. Productivity is measured by a single item WHO General Health Questionnaire and Performance at Work [27]. In this, participants should assign a score (0–10) for their labor productivity over the past three months.

### Cost-effectiveness

The cost-effectiveness of interventions will be calculated according to the cost effectiveness incremental [28,29]. For this, we considered the cost required to conduct ergonomic guidelines and cost guidelines in conjunction with job rotation. These values are divided by the time lost from work.

### Statistical analysis

Descriptive statistics (frequencies, means, standard deviation, standard error, confidence interval) will be used in the analysis of socio-demographic characteristics of the population. The Kolmogorov Smirnov test is used to assess the normality of the data. The chi-square test is used to evaluate the blinding of the assessor by comparing the randomization codes and the evaluator. The difference between the groups and their respective confidence intervals are calculated using linear mixed models [30]. The significance level is 5%. The statistical program SPSS will be used for all analyses, which will be held following the principles of intention to treat.

## Discussion

This study design was developed to investigate the effectiveness of job rotation to prevent musculoskeletal disorders in industrial workers. Despite being a topic of great relevance, due to the difficulty of completely reducing the risk factors of the work environment, few studies have evaluated the effect of job rotation in reducing absenteeism caused by musculoskeletal disorders. It is expected that interventions to reduce the occurrence of musculoskeletal disorders and the consequent removal of the workers for sick leave, 100 hours of lost work. It is also hoped that the results obtained in this study may contribute to setting standards in the field of Occupational Health, as well as for decision making of professionals working in this area.

## Competing interests

The authors declare that they have no competing interests.

## Authors’ contributions

MLCC and RSP were responsible for designing the study. RSP procured funding. All authors have contributed to the manuscript. All authors read and approved the final manuscript.

## Pre-publication history

The pre-publication history for this paper can be accessed here:

http://www.biomedcentral.com/1471-2474/15/170/prepub
